# Combined Nivolumab and Ipilimumab Therapy Promotes Immune‐Mediated Cardiomyocyte Apoptosis Through TLR4–Myd88–NF‐*Κ*b–Driven Activation of the NLRP3 Inflammasome

**DOI:** 10.1155/humu/8506248

**Published:** 2026-04-23

**Authors:** Mengjiao Gao, Xinya Liu, Li Wu, Yun Jiang, Jian Yu, Yuanming Zhang

**Affiliations:** ^1^ Xinjiang Medical University Affiliated Tumor Hospital (The Third Clinical Medical College), Urumqi City, Xinjiang Uygur Autonomous Region, China; ^2^ The Fifth Affiliated Hospital of Xinjiang Medical University, Urumqi City, Xinjiang Uygur Autonomous Region, China, xjmu.edu.cn

**Keywords:** *Caulis sinomenii*, CTLA-4, CV-irAE, PD-1, TLR4

## Abstract

Immune checkpoint inhibitors (ICIs) have revolutionized cancer treatment but are associated with serious immune‐related cardiac toxicities. In this study, AC16 human cardiomyocytes were exposed to nivolumab alone or in combination with ipilimumab. The expression of apoptosis‐ and inflammation‐associated proteins and key components of the TLR4–MyD88–NF‐*κ*B signaling pathway was examined by Western blotting. Apoptotic responses were evaluated using flow cytometry and immunofluorescence assays. BALB/c mice were administered nivolumab or nivolumab plus ipilimumab intraperitoneally for 4 weeks followed by histopathological assessment of cardiac tissue and measurement of myocardial injury biomarkers. Network pharmacology, protein–protein interaction analysis, GO/KEGG enrichment, and molecular docking were applied to identify active constituents of *Caulis sinomenii* and their potential targets in immune‐related cardiac injury. Combined treatment with nivolumab and ipilimumab markedly increased cardiomyocyte apoptosis and elevated the expression of NLRP3 and ASC. Increased phosphorylation of IKK*β* and NF‐*κ*B p65 indicated activation of the TLR4–MyD88–NF‐*κ*B signaling cascade. Knockdown of either TLR4 or NLRP3 significantly mitigated apoptosis and reduced inflammatory protein expression. In vivo, combined ICI therapy led to higher levels of myocardial injury markers and proinflammatory mediators. Network pharmacology analysis identified six major compounds from *Caulis sinomenii*, with sinomenine showing strong predicted binding to TLR4 (binding energies ranging from −9.6 to −5.7 kcal/mol). These findings demonstrate that under the present experimental conditions, combined nivolumab and ipilimumab treatment was associated with greater activation of the TLR4–MyD88–NF‐*κ*B–NLRP3 axis and greater cardiomyocyte injury than nivolumab alone. However, because the combination group received a higher total antibody dose, the current study does not distinguish whether this difference reflects increased total drug exposure, additivity, or the specific contribution of CTLA‐4 blockade. Bioactive constituents of *Caulis sinomenii*, particularly sinomenine, may represent potential cardioprotective modulators against ICI‐induced cardiac injury.

**Highlight**

Dual immunotherapy drugs, nivolumab combined with ipilimumab was associated with greater myocardial injury than nivolumab alone in our models.

In vivo and in vitro models have confirmed that nivolumab combined with ipilimumab affects myocardial apoptosis by regulating the Nod‐like receptor Protein 3 (NLRP3) inflammasome through the TLR4–MyD88–NF‐*κ*B signaling pathway.

Investigating the molecular mechanism of *Caulis sinomenii* in treating immune‐related myocardial injury through network pharmacology, molecular docking, and toll‐like Receptor 4 (TLR4) was suggested as a potential target.

This study provides new insights for patients with myocardial injury based on tumor immunotherapy and offers novel therapeutic targets for clinical use.

## 1. Introduction

Immune checkpoint inhibitors (ICIs) are antibody‐based therapies that block immune checkpoint molecules such as CTLA‐4, PD‐1, and PD‐L1, thereby enhancing antitumor immune responses [1–3]. ICIs activate the immune system at the same time, with antitumor off‐target effects possibly resulting in immune‐related adverse reactions to other organs (immune‐related adverse events [irAEs]). Although the incidence of immune‐related cardiotoxicity is low, the mortality rate is high [4, 5], and myocarditis is the most common manifestation [6]; however, some severe patients may present with acute heart failure or even rapid progression to cardiogenic shock [7]. The clinical manifestations of ICI‐related cardiotoxicity can vary [8, 9]. Myocarditis is the most common type of infection [6]. Myocarditis most commonly occurs a median of 17–34 days after the start of ICIs treatment, but some patients do not develop myocarditis until several months after ICIs treatment [10]. The electrocardiography (ECG) of most patients with myocarditis can reveal various manifestations, such as prolonged QT intervals, inverted T waves, and abnormal Q waves [11]. Nearly three‐quarters of patients with myocarditis have elevated troponin (cTn) levels, and the increase in cardiac troponin T (TnT) levels is more significant in symptomatic patients than in asymptomatic patients. Other indicators, such as B‐type natriuretic peptide (BNP), N‐terminal B‐type natriuretic peptide (NT‐proBNP), the erythrocyte sedimentation rate (ESR), C‐reactive protein, and autoimmune antibodies, are also helpful in the diagnosis of heart injury. At present, there is no clear epidemiological evidence for risk factors affecting immune‐related cardiotoxicity.

Electrocardiogram, Echocardiography, or cardiac magnetic resonance (CMR) can be performed in patients with suspected ICI‐related cardiotoxicity [12]. A decrease in overall longitudinal strain (GLS) may occur in the early stages of myocarditis [13, 14]. CMR imaging is the gold standard for the noninvasive diagnosis of myocarditis [15, 16]. ICI‐related cardiotoxic injuries often progress rapidly, and treatment should be initiated as soon as possible once a patient is diagnosed [17]. Current initial treatments include immediate discontinuation of ICIs and the use of glucocorticoids [18, 19]. If there is no significant improvement or clinical deterioration, second‐line immunosuppression should be considered [20, 21]. There are a few prospective studies on the treatment of ICI‐related cardiotoxicity. Owing to existing experience and limited evidence, the cessation of ICIs therapy and the initiation of immunosuppressive therapy are still the cornerstones of the treatment of ICI‐related cardiotoxicity [22]. In clinical practice, hormone therapy and symptomatic supportive therapy are often selected at the same time [19, 23].

Therefore, cardiovascular problems have become important factors affecting the survival and prognosis of cancer patients. The pathogenesis of ICI‐related cardiotoxicity has become urgent, but the pathogenesis of ICI‐related cardiotoxicity remains unclear [24]. Some experts and scholars have speculated that this phenomenon is related to the disruption of the balance between autoimmunity and immune tolerance in the body [25]. In addition, studies have suggested that the expression of common antigens between the myocardium and tumor tissues may be one of the mechanisms of ICI‐related cardiotoxicity [26]. Previous studies have shown that the mechanisms of tumor‐related cardiotoxicity include mitochondrial dysfunction and reactive oxygen‐induced oxidative stress, lysosome damage, autophagy damage, cellular senescence, DNA damage, and NLRP3 inflammasome formation and activation [27]. In clinical cases, when heart injury occurs in patients with tumors after immunotherapy, the clinical manifestation is a relatively severe inflammatory response, and even inflammatory factor storms are generated. Therefore, we investigated whether the mechanism of immunotherapy‐related heart injury is related to the expression of the inflammatory body NLRP3.

The NLRP3 inflammasome is involved in the onset and progression of various diseases [28]. NLRP3 inflammasome activation is classically associated with inflammatory cell death pathways, including pyroptosis, but may also be linked to apoptotic signaling under certain conditions [29]. The priming and activation of the NLRP3 inflammasome are closely related to toll‐like Receptor 4 (TLR4), the adaptor molecule myeloid differentiation Factor 88 (MyD88), and the downstream transcription factor nuclear factor kappa B (NF‐*κ*B). Therefore, the TLR4/MyD88/NF‐*κ*B signaling pathway is essential for the activation of the NLRP3 inflammasome. The NF‐*κ*B nuclear transcription factor plays an important role in regulating the immune response and inflammation. It is involved in the expression of genes involved in many processes that play key roles in the development and progression of cancer, such as proliferation, migration, and apoptosis [30]. In addition, MyD88, a central node of the inflammatory pathway, is widely expressed in diseases of the immune and cardiovascular systems [31]. Studies have shown that the TLR4/NF‐*κ*B signaling pathway may be involved in doxorubicin‐induced cardiotoxicity [32]. Therefore, we are aimed at exploring the role of the TLR4–MyD88–NF‐*κ*B signaling pathway and the NLRP3 inflammasome in ICI‐related myocardial injury to identify effective targets for patients with immune‐related cardiotoxicity.

Traditional Chinese medicine (TCM) has been widely recognized in the fields of cardiovascular disease and cancer treatment and has great potential in preventing and treating cardiotoxicity caused by antitumor drugs [33, 34]. It includes single herbs, herbal compounds, Chinese patent medicines, and herbal extracts, such as astragalus, Sini decoction, Shengmai beverage, Shengmai injection, Tongxinluo capsule, Yangxinxin granule, rutin, and curcumin, and so on, all of which have shown significant preventive effects and therapeutic effects against cardiotoxicity caused by tumor drugs [35, 36]. Overall, this evidence highlights the potential of TCM therapy as an effective way to reduce myocardial toxicity induced by antitumor drugs. However, identifying the active ingredients in Chinese medicines is still the subject of further research.

## 2. Materials and Methods

### 2.1. Drugs and Reagents

AC16 human myocardial cells were purchased from Cybertron (Shanghai) Biotechnology Co, Ltd. (Product Number iCell‐h323). The nivolumab (anti‐PD‐1) PD‐1 inhibitor was purchased from MCE (United States) Biotechnology Company, Product Number HY‐P9903; the ipilimumab (anti‐CTLA‐4) CTLA‐4 inhibitor was purchased from MCE (United States) Biotechnology Company, Product Number HY‐P9901; the si‐TLR4 inhibitor was purchased from Guangzhou Ruibo; and the si‐NLRP3 inhibitor was purchased from Guangzhou Ruibo. Bax, which cleaved caspase‐1, IL‐1*β*, an inhibitor of NF‐*κ*B*α* (I) *κ*B*α*, phospho‐I*κ*B*α* (Ser32), I*κ*B kinase *β* (IKK*β*), phospho‐IKK*α*/*β* (Ser176/180), NF‐*κ*B p65, and phospho‐NF‐*κ*B p65 (Ser536) antibodies (Cell Signaling Technology), and Bcl‐2, ASC, caspase‐1, NLRP3, IL‐18, TLR4, and Myd88 antibodies were purchased from Abcam Company in the United States. No isotype‐matched or nonspecific IgG control antibodies were included in the present study.

### 2.2. Cell Grouping and Treatment

AC16 human cardiomyocytes were divided into three groups according to different treatments: the control group (untreated cells without antibody exposure), the monotherapy group (treated with the PD‐1 inhibitor nivolumab at a concentration of 10 *μ*g/L), and the combined treatment group (treated with the PD‐1 inhibitor nivolumab at 10 *μ*g/L together with the CTLA‐4 inhibitor ipilimumab at 5 *μ*g/L). Cells were exposed to the indicated treatments for 24 h before subsequent analyses.

### 2.3. Animal Grouping and Management

Male BALB/c mice (6–8 weeks old, 20–24 g) were housed under standard laboratory conditions at a controlled temperature of 22^°^C ± 2^°^C, relative humidity of 50*%* ± 5*%*, and a 12‐h light–dark cycle, with ad libitum access to food and water. All animal procedures were performed in compliance with the Guide for the Care and Use of Laboratory Animals (National Institutes of Health, United States) and approved by the Animal Testing Ethics Committee of Xinjiang Medical University Affiliated Tumor Hospital (The Third Clinical Medical College), Xinjiang, Urumqi City, China (Ethics Approval Number MDL2023‐10‐20‐03). BALB/c mice were randomly divided into 3 groups (*n* = 5 in each group): (1) control group (saline‐treated animals without antibody exposure), (2) the monotherapy group (PD‐1 inhibitor/nivolumab), and 3) the combination group (PD‐1 inhibitor combined with CTLA‐4 inhibitor/nivolumab combined with ipilimumab). The control group was injected with normal saline, the monotherapy group was injected intraperitoneally (i.p) with the PD‐1 inhibitor nivolumab at a dose of 5 mg/kg, and the combined treatment group was injected i.p with nivolumab (5 mg/kg) together with ipilimumab (5 mg/kg), for a total antibody dose of 10 mg/kg. Once every 3 days for 4 weeks, at the end of the experiment, the mice were anesthetized (4% [*w*/*v*]) sodium pentobarbital, 40 mg/kg i.p), and the myocardial tissues were quickly collected and stored at −80°C.

### 2.4. Western Blot Analysis

AC16 human cardiomyocytes were cultured in DMEM/F‐12 medium supplemented with 10% fetal bovine serum (FBS) and maintained at 37°C in a humidified incubator with 5% CO_2_. For Western blot analysis, the blank group refers to untreated AC16 cardiomyocytes cultured under identical conditions without drug exposure or siRNA transfection, serving as the baseline control. Cells treated with ICIs or transfected with si‐TLR4, si‐NLRP3, or negative control siRNA were compared against this blank control unless otherwise specified. Samples were homogenized on ice prior to lysis. Protein concentrations were determined using a BCA protein assay kit according to the manufacturer′s instructions. Equal amounts of protein (20–40 *μ*g) were separated by sodium dodecyl sulfate–polyacrylamide gel electrophoresis (SDS‐PAGE) and subsequently transferred onto polyvinylidene difluoride (PVDF) membranes.

Membranes were blocked with 5% nonfat milk or bovine serum albumin (BSA) in Tris‐buffered saline containing 0.1% Tween‐20 (TBST) for 1 h at room temperature and then incubated overnight at 4°C with primary antibodies against BAX, Bcl‐2, cleaved caspase‐3, caspase‐3, NLRP3, ASC, caspase‐1, IL‐1*β*, IL‐18, TLR4, MyD88, IKK*β*, phospho‐IKK*β* (Ser176/180), NF‐*κ*B p65, phospho‐NF‐*κ*B p65 (Ser536), and *β*‐actin. After washing with TBST, membranes were incubated with horseradish peroxidase–conjugated secondary antibodies for 1 h at room temperature. Protein bands were visualized using enhanced chemiluminescence (ECL) reagents and quantified by densitometric analysis using ImageJ software, with *β*‐actin serving as the internal loading control.

### 2.5. Cell Viability Assay

AC16 human cardiomyocytes were seeded into 96‐well plates and cultured in Gibco DMEM/F‐12 medium (Cat. No. 11320033) supplemented with 10% FBS (Cat. No. 10099141) and 1% penicillin–streptomycin (Cat. No. 15140122) at 37°C in a humidified incubator with 5% CO_2_. After overnight attachment, cells were treated with nivolumab alone or in combination with ipilimumab for 24 h. Cell viability was then assessed using PrestoBlue Cell Viability Reagent (Thermo Fisher Scientific, Cat. No. A13261) according to the manufacturer′s instructions. Briefly, PrestoBlue reagent was added directly to each well, the plate was incubated for 10 min at 37°C, and absorbance was measured using a microplate reader. Cell viability was expressed as a percentage of the untreated control group. PrestoBlue is a ready‐to‐use, resazurin‐based reagent compatible with absorbance‐ or fluorescence‐based microplate reading and is intended for rapid viability measurement in mammalian cells.

### 2.6. FCM (Flow Cytometry)

Apoptosis of AC16 cardiomyocytes was assessed using an Annexin V‐FITC/propidium iodide (PI) apoptosis detection kit according to the manufacturer′s protocol. Briefly, following drug treatment, cells were harvested, washed twice with cold phosphate‐buffered saline (PBS), and resuspended in binding buffer. Cells were then incubated with Annexin V‐FITC and PI for 15 min at room temperature in the dark. Stained cells were analyzed using a FCM, and data were processed using FlowJo software. Early apoptotic cells were defined as Annexin V‐positive/PI‐negative, whereas late apoptotic or necrotic cells were Annexin V‐positive/PI‐positive.

### 2.7. Immunofluorescence Staining

For immunofluorescence staining, AC16 cardiomyocytes were seeded onto coverslips and subjected to the indicated treatments. Cells were fixed with 4% paraformaldehyde for 15 min at room temperature, permeabilized with 0.1% Triton X‐100 for 10 min, and blocked with 5% BSA for 1 h. Subsequently, cells were incubated overnight at 4°C with primary antibodies against apoptosis‐ or inflammation‐related proteins. After washing with PBS, cells were incubated with Alexa Fluor 488– or Alexa Fluor 594–conjugated secondary antibodies for 1 h at room temperature in the dark. Nuclei were counterstained with DAPI. Fluorescence images were captured using a fluorescence microscope, and signal intensity was quantified using ImageJ software.

### 2.8. Immunohistochemistry

Mouse heart tissues were fixed in 10% neutral‐buffered formalin, embedded in paraffin, and sectioned at a thickness of 4 *μ*m. Sections were deparaffinized in xylene and rehydrated through a graded ethanol series. Antigen retrieval was performed using citrate buffer (pH 6.0) under heat‐mediated conditions. Endogenous peroxidase activity was blocked using 3% hydrogen peroxide, followed by blocking with normal serum. Tissue sections were incubated overnight at 4°C with primary antibodies against BNP and cardiac TnT. After washing, sections were incubated with HRP‐conjugated secondary antibodies and developed using diaminobenzidine (DAB) substrate. Nuclei were counterstained with hematoxylin. Stained sections were observed under a light microscope, and positive staining areas were semiquantitatively analyzed using ImageJ software.

### 2.9. Statistical Analysis

GraphPad Prism 9.5 software was used for statistical analysis and plotting. All data are presented as mean ± SD. Comparisons between two groups were performed using unpaired Student′s *t*‐test. Comparisons among three or more groups were performed using one‐way analysis of variance (ANOVA) followed by Dunnett′s post hoc test for multiple comparisons. A *p* value < 0.05 was considered statistically significant. For in vitro experiments, data were obtained from at least three independent biological replicates unless otherwise specified. For animal experiments, each group contained five mice (*n* = 5). The statistical method and sample size used for each experiment are also indicated in the corresponding figure legends.

### 2.10. Construction of the “Active Ingredient–Pathway–Target” Network

#### 2.10.1. Screening of the Active Ingredients of *Caulis Sinomenii*

The active ingredients of *Schisandra chinensis* were screened via the TCM Systematic Pharmacology Database and Analysis Platform (TCMS). Oral bioavailability (OB) ≥ 30*%* and a drug − like (DL) coefficient ≥ 0.1 were used as search criteria to obtain the active components of *Caulis sinomenii*. All the components were imported into the SwissTargetPrediction database by obtaining Smiles numbers through the PubChem database, and the species were defined as “*Homo sapiens*” and “probability ≥ 0.1” to predict the targets of the components.

#### 2.10.2. Screening of Key Components of Disease

“Immune‐related myocardial injury” was used as the keyword, the gene targets related to immunotherapy‐related myocardial injury were entered into the GeneCards, OMIM, and other databases, and the relevance score of GeneCard was set to ≥ 1 as the search condition. The disease targets of the above database were subsequently summarized via Excel software, and the duplicate genes were deleted to construct a database of immunotherapy‐related myocardial injury targets. Finally, the key targets associated with the intersection of TCM and diseases were identified via the Venny 2.1.0 website.

#### 2.10.3. Construction of a Protein–Protein Interaction (PPI) Network Diagram

The intersection targets of *Caulis sinomenii* and immunotherapy‐related myocardial injury were entered into the “multiple proteins” module in the STRING database, the species was set to human (*H*. *sapiens*), the PPI network TSV file was obtained, the TSV file was imported into Cytoscape software 3.9.1, and the CytoHubba plug‐in was used to analyze the PPI network of the key targets.

#### 2.10.4. Gene Function and Pathway Enrichment Analysis

The intersection target of *Caulis sinomenii* and immunotherapy‐related myocardial injury was input into DAVID, the species was set to human (*H*. *sapiens*), and gene ontology (GO) biological enrichment analysis and Kyoto Encyclopedia of Genes and Genomes (KEGG) pathway enrichment analysis of key targets were performed. *p* < 0.01 was used as the screening criterion, and the Top 10 items in the GO analysis of biological process (BP), cell component (CC), and molecular function (MF), and the Top 20 items in the KEGG pathway analysis were included in the next step of the analysis.

#### 2.10.5. Cytoscape Visualization of the “Drug‐Component‐Target‐Pathway” Network

The active ingredient obtained in Item 2.9.1 and the most significant pathway under Item 2.9.4 are mapped to the obtained intersecting targets, and the “drug‐component‐target‐pathway” network diagram is constructed via Cytoscape 3.9.1 software. The intersecting targets are analyzed again, and the interaction relationships between the active ingredients and the intersecting targets and pathways are displayed.

#### 2.10.6. Molecular Docking Between Active Ingredients and Key Targets

The core target with the highest degree value in the PPI network was selected as the receptor, and the active ingredients of *Caulis sinomenii* were selected as ligands for molecular docking analysis. The three‐dimensional crystal structure of TLR4 was obtained from the Protein Data Bank (PDB ID: 3FXI). Prior to docking, the receptor structure was prepared by removing water molecules and heteroatoms, and hydrogen atoms were added. The chemical structures of the active ingredients were retrieved from the PubChem database, and ligand structures were subjected to energy minimization to obtain stable conformations before docking. Molecular docking was performed using standard protocols, and binding affinity was evaluated based on binding energy values. PyMOL software was used to visualize the docking conformations of the key active ingredients with the core target protein TLR4, highlighting hydrogen bonds and hydrophobic interactions between the ligands and the receptor.

## 3. Results

### 3.1. Effects of Nivolumab Alone or Combined With Ipilimumab on Apoptosis‐ and Inflammation‐Related Protein Expression in AC16 Human Cardiomyocytes

**Figure 1:**
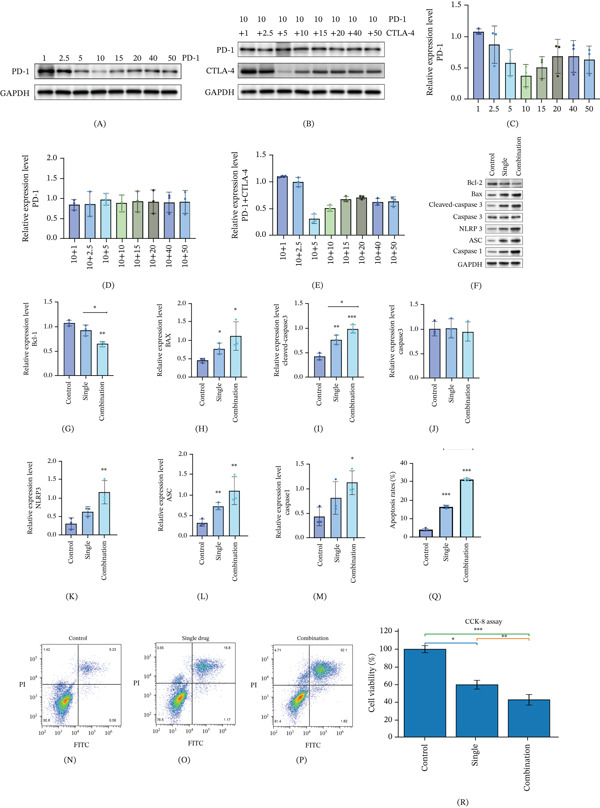
Nivolumab alone or combined with ipilimumab promotes apoptosis and inflammatory responses in AC16 cardiomyocytes. (A, B) Western blot analysis used to determine appropriate concentrations of nivolumab based on the expression of inflammation‐related proteins (NLRP3, ASC, caspase‐1, IL‐18, and IL‐1*β*) in AC16 cardiomyocytes. (C–E) Quantification of apoptosis‐related proteins BAX, Bcl‐2, and cleaved caspase‐3 following treatment with nivolumab alone or in combination with ipilimumab. (F) Representative Western blot showing apoptosis‐related protein expression. (G–J) Quantitative analysis of Bcl‐2, BAX, cleaved caspase‐3, and caspase‐3 expression levels. (K–M) Expression of inflammation‐related proteins (NLRP3, ASC, and caspase‐1) detected by Western blot. (N–Q) Flow cytometry analysis of apoptosis in AC16 cardiomyocytes using Annexin V‐FITC/PI staining. Data are presented as mean ± SD from three independent biological replicates (*n* = 3). (R) Cell viability assessed using the PrestoBlue assay following treatment with nivolumab alone or in combination with ipilimumab. Statistical significance was determined using one‐way ANOVA followed by Dunnett′s post hoc test for multiple comparisons. *p* < 0.05;  ^∗^ *p* < 0.01;  ^∗∗^ *p* < 0.001; ns, not significant.

To evaluate the effects of ICIs on cardiomyocyte injury, AC16 human cardiomyocytes were treated with nivolumab alone or in combination with ipilimumab, and apoptosis‐ and inflammation‐related protein expression was analyzed by Western blotting. As shown in Figure 1A,B, nivolumab treatment was associated with concentration‐dependent changes in protein expression, and concentrations producing measurable but nonsaturating effects were selected for subsequent experiments. To further validate the appropriateness of the selected concentrations, cell viability and apoptosis were assessed. As shown in Figure 1R, nivolumab treatment reduced cell viability compared with the control group, and this effect was more pronounced in the combination treatment group. Compared with the control group, nivolumab monotherapy increased the expression of proapoptotic proteins, including BAX and cleaved caspase‐3, while decreasing the expression of the antiapoptotic protein Bcl‐2 (Figure 1C–E). Notably, combined treatment with nivolumab and ipilimumab resulted in a more pronounced increase in BAX and cleaved caspase‐3 expression and a greater reduction in Bcl‐2 levels compared with nivolumab alone. These results indicate that treatment with nivolumab, particularly in combination with ipilimumab, was associated with increased expression of apoptosis‐related proteins in AC16 cardiomyocytes.

### 3.2. Nivolumab Combined With Ipilimumab Enhances Cardiomyocyte Apoptosis and Inflammation

The mechanism of cardiac toxicity related to tumor therapy includes the formation and activation of the NLRP3 inflammasome, as well as the mechanism of action and cell death. On the basis of the above experimental results, we continued to carry out myocardial intracellular experiments. We detected the expression of apoptosis‐related factors (Figure 1F). Through Western blot, nivolumab combined with ipilimumab weakened the expression of Bcl‐2 apoptosis inhibitory molecules (Figure 1G), increased the expression of cleaved caspase‐3 and BAX (Figure 1H,I) and did not affect the expression of caspase‐3 (Figure 1J). Nivolumab enhances myocardial cell apoptosis, and the effect of nivolumab combined with ipilimumab on myocardial apoptosis is more significant than that of nivolumab alone.

NLRP3, a cytoplasmic supermolecule complex, is composed of NLRP3, ASC, and caspase‐1 and induces interleukin‐1*β*. The secretion and release of IL‐18 promote cell death [21, 37]. The activation of the NLRP3 inflammasome is known to be associated with inflammatory cell death pathways and may be linked to apoptotic signaling. We continued to carry out follow‐up experimental verification. Through Western blot, we determined that the expression of NLRP3 and ASC, which are inflammatory‐related factors of myocardial cells, was increased by nivolumab and nivolumab combined with ipilimumab (Figure 1K,L). Compared with that in the single‐drug group, the effect in the combined drug group was more significant; thus, nivolumab combined with ipilimumab can enhance the inflammation of myocardial cells but has little effect on the expression of caspase‐1 (Figure 1M).

Then, we detected the degree of apoptosis via FCM, one of the classical methods used to detect apoptosis. Compared with that in the control group, the number of apoptotic cells was greater in the single group and the combination group. Moreover, the number of apoptotic cells in the combined group was greater than that in the other groups. (Figure 1N–Q). We verified that combining nivolumab with ipilimumab increased the degree of apoptosis via different methods.

### 3.3. Increased TLR4–Myd88–NF‐*κ*B Pathway Expression in Combination With Nivolumab and Ipilimumab

**Figure 2:**
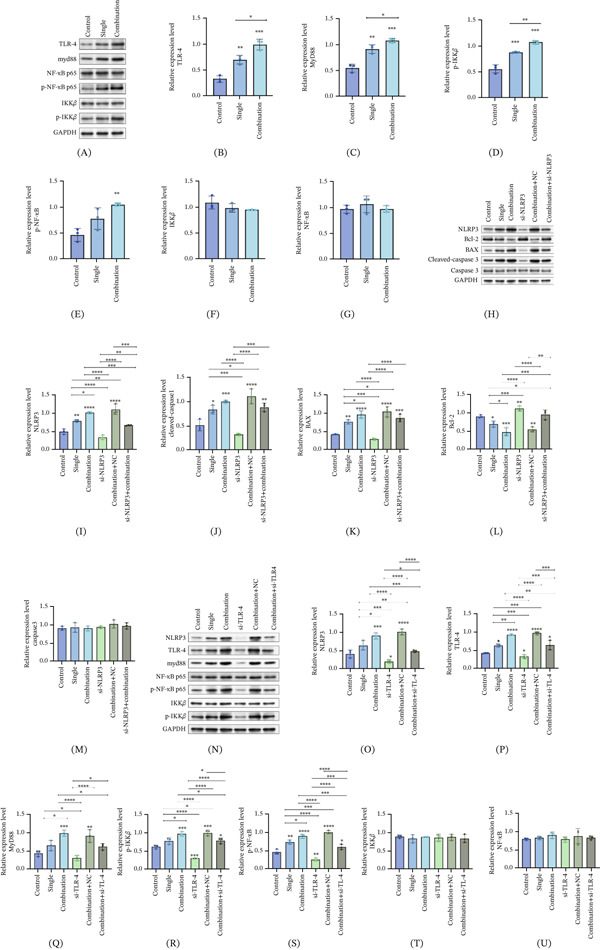
Nivolumab combined with ipilimumab activates the TLR4–MyD88–NF‐*κ*B pathway, whereas TLR4 or NLRP3 knockdown attenuates apoptosis‐related changes in AC16 cardiomyocytes. (A) The expression of TLR4–MyD88–NF‐*κ*B pathway‐related proteins was detected via Western blot. (B–G) The protein expression levels of TLR4, MyD88, NF‐*κ*B, p‐NF‐*κ*B, IKK*β*, and p‐IKK*β*. Single‐drug group: PD‐1, programmed cell death Protein 1; combination drug group: CTLA‐4, cytotoxic T lymphocyte‐associated antigen‐4; NF‐*κ*B, nuclear factor kappa‐B; TLR4, Toll‐like Receptor 4. (H) Representative Western blot showing the effects of TLR4 or NLRP3 silencing on apoptosis‐related protein expression in AC16 cardiomyocytes treated with nivolumab combined with ipilimumab. (I–M) Quantification of apoptosis‐related proteins, including NLRP3, Bcl‐2, BAX, cleaved caspase‐3, and caspase‐3. (N) Representative Western blot showing the effects of TLR4 silencing on NLRP3 inflammasome components and TLR4–MyD88–NF‐*κ*B pathway‐related proteins. (O–U) Quantification of NLRP3, TLR4, MyD88, NF‐*κ*B, p‐NF‐*κ*B, IKK*β*, and p‐IKK*β* expression levels. Data are presented as mean ± SD from three independent biological replicates (*n* = 3). Statistical significance was determined using one‐way ANOVA followed by Dunnett′s post hoc test for multiple comparisons. *p* < 0.05;  ^∗^ *p* < 0.01;  ^∗∗^ *p* < 0.001; ns, not significant.

Numerous studies have suggested that the classic pathway of NLRP3 inflammasome initiation is related to TLR4/Myd88/NF‐*κ*B. We determined through Western blot that the combination of nivolumab and ipilimumab enhances the expression of TLR4, Myd88, p‐IKK*β*, and p‐NF‐*κ*B p65 (Figure 2A–E). However, for IKK*β* and NF‐*κ*B, p65 had little effect (Figure 2F,G). Compared with nivolumab, the combination of nivolumab and ipilimumab had a more significant effect on TLR4. We found through cell experiments that the combination of nivolumab and ipilimumab enhances TLR4–Myd88–NF‐*κ*B pathway expression.

### 3.4. Knockdown of TLR4 or NLRP3 Attenuates Nivolumab Plus Ipilimumab‐Associated Cardiomyocyte Apoptosis by Inhibiting the TLR4–MyD88–NF‐*κ*B Signaling Pathway

To determine whether the TLR4–MyD88–NF‐*κ*B–NLRP3 signaling axis mediates cardiomyocyte injury induced by nivolumab combined with ipilimumab, siRNA‐mediated knockdown of NLRP3 or TLR4 was performed in AC16 cells. Western blot analysis demonstrated that silencing of NLRP3 or TLR4 significantly attenuated cardiomyocyte apoptosis associated with the combined ICI treatment (Figure 2H). Specifically, the expression levels of proapoptotic proteins, including NLRP3, cleaved caspase‐3, and BAX, were markedly reduced following knockdown (Figure 2I–K), whereas the expression of the antiapoptotic protein Bcl‐2 was significantly increased (Figure 2L). In contrast, total caspase‐3 expression remained largely unchanged (Figure 2M). Furthermore, pathway‐related proteins were examined to elucidate the underlying mechanism. Compared with the combined ICI‐treated group, TLR4 or NLRP3 knockdown markedly suppressed activation of the NLRP3 inflammasome and the TLR4–MyD88–NF‐*κ*B signaling pathway, as evidenced by reduced expression of NLRP3, TLR4, MyD88, phosphorylated IKK*β*, and phosphorylated NF‐*κ*B p65 (Figure 2N–S). Notably, the total protein levels of IKK*β* and NF‐*κ*B p65 were not significantly altered (Figure 2T,U), indicating that the inhibitory effects were primarily exerted at the level of pathway activation rather than total protein expression.

### 3.5. Nivolumab Combined With Ipilimumab Enhances the Apoptosis and Inflammation of Myocardial Cells in Mice

**Figure 3:**
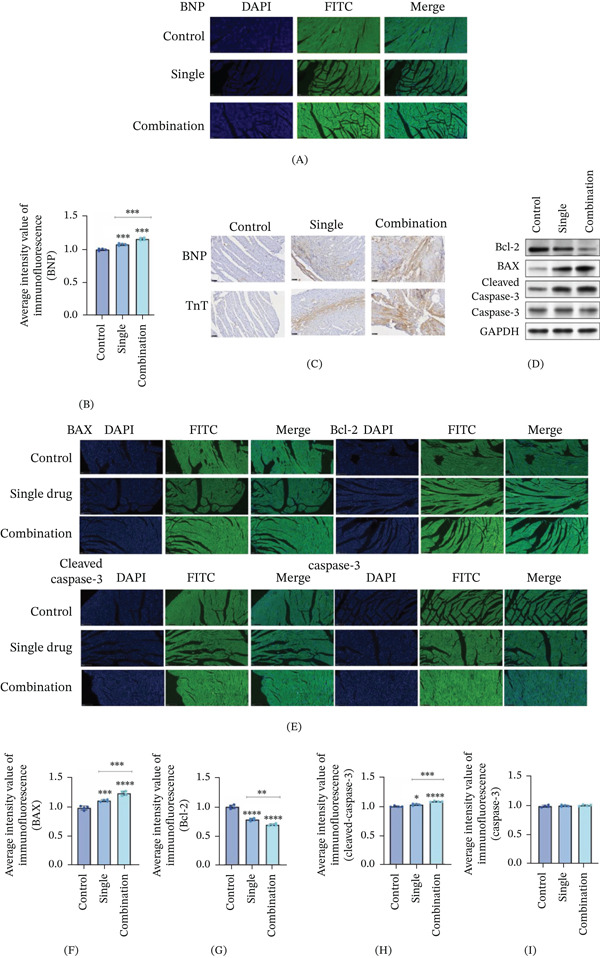
Nivolumab combined with ipilimumab enhanced the degree of apoptosis and inflammation in mouse cardiomyocytes. (A, B) The expression of the myocardial injury factor BNP was detected via immunofluorescence staining. (C) The expression of the myocardial injury factors BNP and TnT was detected by immunohistochemistry. (D–I) The expression of the cardiac apoptosis‐related proteins BAX, Bcl‐2, cleaved caspase‐3, and caspase‐3 was detected via immunofluorescence staining and Western blot. Single group: PD‐1, programmed cell death Protein 1; combination group: CTLA‐4, cytotoxic T lymphocyte‐associated antigen‐4. Data are presented as mean ± SD. Each group consisted of five biological replicates (mice) (*n* = 5). Statistical significance was determined using one‐way ANOVA followed by Dunnett′s post hoc test for multiple comparisons. *p* < 0.05;  ^∗^ *p* < 0.01;  ^∗∗^ *p* < 0.001; ns, not significant.

We demonstrated that the combination of nivolumab and ipilimumab promoted cardiomyocyte apoptosis in vitro, and we subsequently performed the same experiment in mice. The expression of myocardial injury factors in the myocardial tissue of the mice was detected via immunofluorescence staining. Compared with that in the control group, the expression of BNP in the myocardial tissue of the mice in both the single group and the combination group was significantly greater (Figure 3A,B). Subsequent immunohistochemical staining revealed that the expression levels of BNP and TnT were significantly greater in both the single group and the combination group than in the control group (Figure 3C). The expression level in the combined treatment group was greater than that in the monotherapy group under the tested dosing conditions; however, because the combination group received a higher total antibody dose, this comparison does not establish a dose‐dependent relationship or distinguish between additivity and increased total drug exposure. After that, we detected the expression of apoptosis‐related proteins (Figure 3D,E). The results were similar. Compared with the control group, both the single‐drug group and the combined‐drug group were associated with increased apoptosis‐related changes in mouse cardiomyocytes, and the combined treatment group showed greater effects than the monotherapy group under the experimental conditions (Figure 3F–I). However, this difference should be interpreted cautiously because the combination group received a higher total antibody dose.

### 3.6. Nivolumab Combined With Ipilimumab Enhances the Expression of the TLR4–Myd88–NF‐*κ*B Signaling Pathway and NLRP3 in the Apoptosis of Mouse Cardiomyocytes

**Figure 4:**
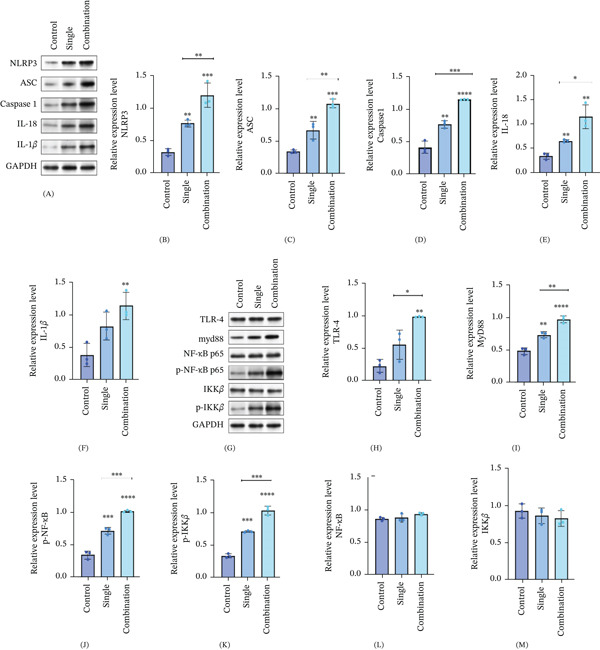
Nivolumab combined with ipilimumab enhances the expression of the TLR4–Myd88–NF‐*κ*B signaling pathway and NLRP3 in the apoptosis of mouse cardiomyocytes. (A) Western blot analysis of the expression of inflammatory factors in the myocardial tissue of mice in the single drug group and combined drug group. (B–F) The expression levels of NLRP3, ASC, caspase‐1, IL‐18 and IL‐1*β*. (G) Western blot analysis of TLR4–MyD88–NF‐*κ*B pathway‐related proteins in the myocardial tissue of mice in the single‐drug group and (H–M) combination drug group, including the protein expression levels of TLR4, Myd88, NF‐*κ*B, p‐NF‐*κ*B, IKK*β*, and p‐IKK*β*. Single‐drug group: PD‐1, programmed cell death Protein 1. The combination drugs used were as follows: CTLA‐4, cytotoxic T lymphocyte–associated antigen‐4; NF‐*κ*B, nuclear factor kappa‐B; TLR4, Toll‐like Receptor 4; and NLRP3, NOD‐like receptor thermal protein domain associated Protein 3. Data are presented as mean ± SD. Each group consisted of five biological replicates (mice) (*n* = 5). Statistical significance was determined using one‐way ANOVA followed by Dunnett’s post hoc test for multiple comparisons. *p* < 0.05;  ^∗^ *p* < 0.01;  ^∗∗^ *p* < 0.001; ns, not significant.

Then, we detected the expression levels of NLRP3‐related inflammatory factors and TLR4–MyD88–NF‐*κ*B–related proteins in mouse myocardial tissue via Western blot. These results are similar to those obtained in vitro, which further validates our conclusion. Compared with those in the control group, the expression levels of the inflammatory cytokines NLRP3, ASC, caspase‐1, IL‐18, and IL‐1*β* in the single‐drug group and the combined‐drug group were significantly greater (Figure 4A–F), and the expression levels of NLRP3 were significantly greater than those of IL‐18 and IL‐1*β*. Moreover, the increase in the combination group was greater than that in the monotherapy group under the tested dosing conditions. We then detected the expression levels of TLR4–MyD88–NF‐*κ*B–related proteins in mouse myocardial tissue (Figure 4G). Compared with those in the control group, the expression levels of TLR4–MyD88–NF‐*κ*B pathway proteins, including TLR4, MyD88, p‐NF‐*κ*B, and p‐IKK*β* (Figure 4H–K), were increased in the single group and combined groups, and the increase in these proteins was greater in the combined group than in the monotherapy group under the tested dosing conditions. However, the expression levels of NF‐*κ*B and IKK*β* did not significantly change (Figure 4L,M). Therefore, nivolumab combined with ipilimumab enhances the expression of the TLR4–MyD88–NF‐*κ*B signaling pathway and NLRP3 in the apoptosis of mouse cardiomyocytes.

### 3.7. Potential Core Target Collection and GO and KEGG Enrichment Analysis of *Caulis Sinomenii*

**Figure 5:**
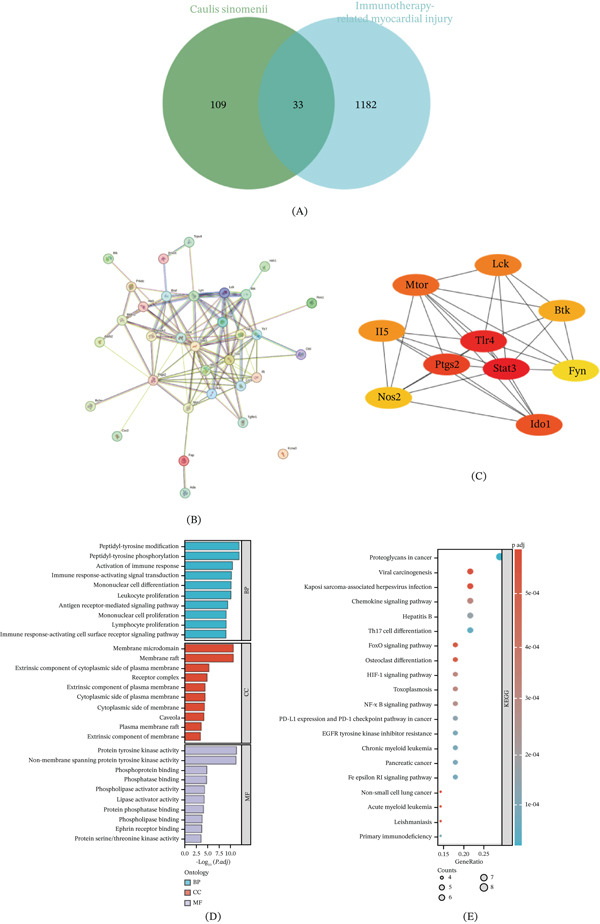
Potential core target collection and GO and KEGG enrichment analyses of Caulis sinomenii. (A) Venn diagram of the common targets of *Caulis sinomenii* and immunotherapy‐associated myocardial injury. **(B)** Core target map of *Caulis sinomenii* in the treatment of the immune‐related myocardial injury. **(C)** Diagram of the Top 10 core targets of the PPI network. **(D)** GO functional enrichment analysis of potential targets of *Caulis sinomenii* in the treatment of immune‐related myocardial injury. **(E)** KEGG enrichment analysis of core targets of *Caulis sinomenii* in the treatment of immune‐related myocardial injury.

In the TCMSP database, 6 active ingredients were obtained, including 16‐epi‐isositsirikine, BSIT, magnograndiolide, michelenolide, sinomenine, and stepholidine, which were predicted to have OB greater than or equal to 30% and DL greater than or equal to 0.1. In the end, in the SwissTarget prediction database,142 constituent targets of *Caulis sinomenii* were predicted. A total of 1215 target genes were obtained via the use of the GeneCards, OMIM, Disgenet, and TTD databases to screen target genes related to immunotherapy‐related myocardial injury and to delete duplicates. Thirty‐three key targets were obtained by intersecting 1215 disease targets with 142 targets of the active ingredient of *Caulis sinomenii* (Figure 5A). A total of 33 intersecting targets were imported into the STRING database for PPI network analysis, the PPI network of common targets was constructed, the free targets were removed, the confidence level was set to 0.4, and the first 10 core targets were obtained via the plug‐in CytoHubba (Figure 5B,C). Prostaglandin‐endoperoxide synthase 2 (PTGS2), TLR4, signal transducer and activator of transcription 3 (STAT3), indoleamine 2,3‐dioxygenase 1 (IDO1), nitric oxide synthase 2 (NOS2), interleukin 5 (IL‐5), mammalian target of rapamycin (mTOR), lymphocyte‐specific protein tyrosine kinase (LCK), Bruton tyrosine kinase (BTK), and FYN proto‐oncogene, Src family tyrosine kinase (FYN). The DAVID online database was subsequently used to perform GO function enrichment analysis of common targets, and 2020 BP items, 56 CC items, and 137 MF items were obtained and sorted by *p* value from small to large, and the Top 10 items were selected for plotting (Figure 5D). The results revealed that BPs included mainly the following: peptidyl‐tyrosine modification, peptidyl‐tyrosine phosphorylation, activation of the immune response, and immune response‐activating signal transduction. CCs include membrane microdomains, membrane rafts, and extrinsic components of the cytoplasmic side of the plasma membrane. MFs mainly reflect protein tyrosine kinase activity, nonmembrane spanning protein tyrosine kinase activity, phosphoprotein binding, phosphatase binding, and protein serine/threonine kinase activity. A total of 117 signaling pathways were obtained from the KEGG pathway analysis, and the Top 20 signaling pathways were selected on the basis of the *p* value. A bubble map was drawn online via microbiometric information (Figure 5E). This pathway is involved mainly in proteoglycans in cancer, viral carcinogenesis, Kaposi sarcoma–associated herpesvirus infection, the chemokine signaling pathway, hepatitis B, Th17 cell differentiation, the FoxO signaling pathway, osteoclast differentiation, HIF‐1 signaling pathway, toxoplasmosis, NF‐kappa B signaling pathway, PD‐L1 expression, and PD‐1 checkpoint pathway in cancer, EGFR tyrosine kinase inhibitor resistance, nonsmall cell lung cancer, acute myeloid leukemia, leishmaniasis, and other related pathways.

### 3.8. “Active Ingredient–Pathway–Target” Network Construction and Molecular Docking

**Figure 6:**
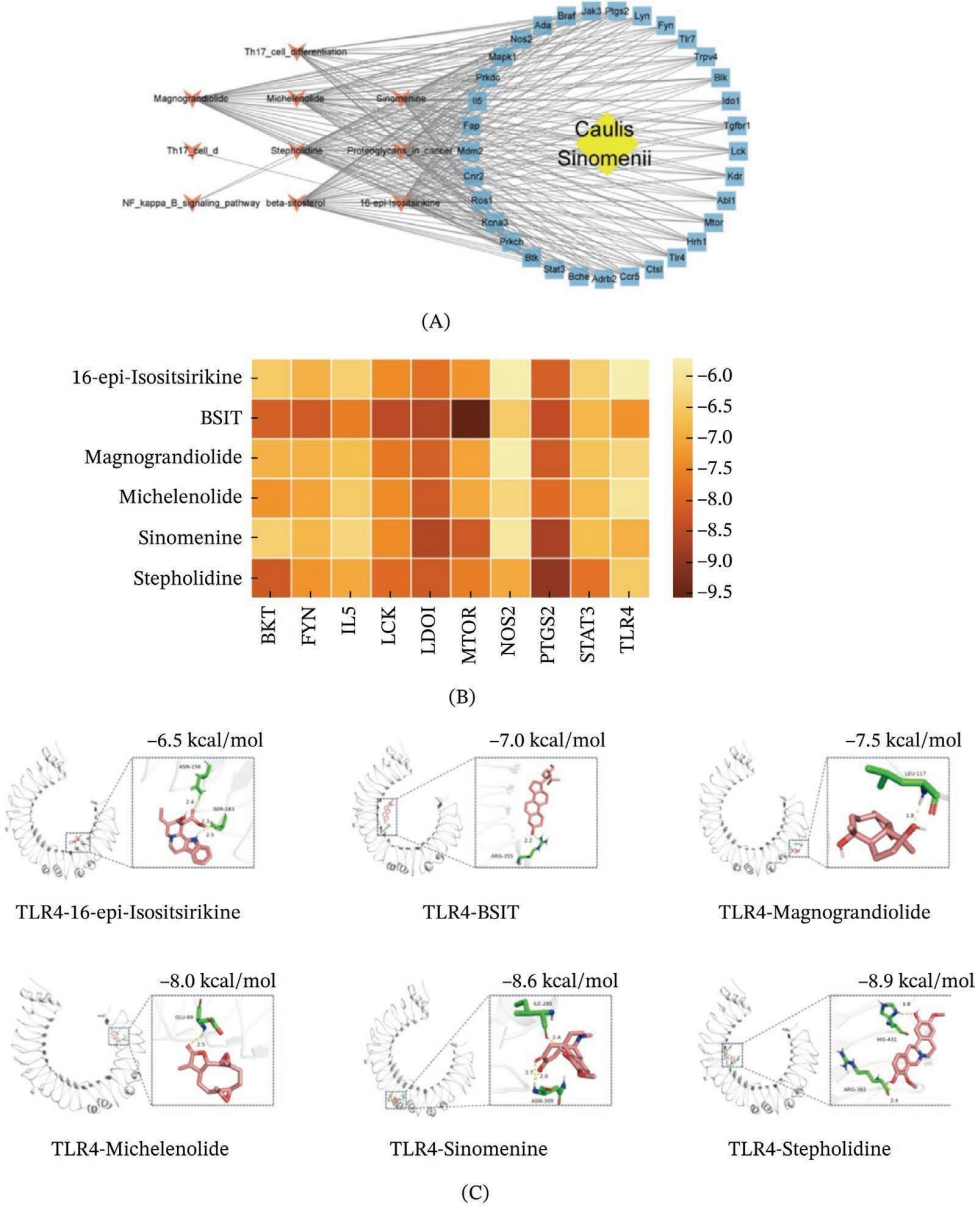
Active ingredient–pathway–target network construction and molecular docking. (A) Construction of the “Caulis sinomenii–active ingredient–pathway–target” network. (B) Molecular docking heatmap of the key active ingredients of *Caulis sinomenii* and the key targets. **(C)** Visualization of the molecular docking of key compounds and core target proteins of *Caulis sinomenii*.

The six active ingredients obtained in the early stage and the most significant Top 20 pathways were mapped to the obtained intersection targets, the “*Caulis sinomenii*–active ingredient–pathway–target” network was established, and the “*Caulis sinomenii*–active ingredient–pathway–target” network was constructed via Cytoscape 3.9.1 software (Figure 6A). The results revealed that PTGS2, TLR4, Stat3, Ido1, NOS2, IL‐5, mTOR, Lck, Btk, and FYN were the core targets of the network, and most of the results overlapped with the results of the first 10 core targets. The 6 active ingredients of 16‐epi‐isositsirikine, BSIT, magnograndiolide, michelenolide, sinomenine, and stepholidine were selected for molecular docking, with 10 target TLR4s obtained via PPI network analysis, and their binding energies were calculated. The results show that the smaller the binding energy of the active ingredient (ligand) and the intersection target (receptor) is, the more stable the conformation, and when the binding capacity of the two is less than −4.25 kcal/mol (1 cal = 4.2 J), it indicates that there is a certain binding capacity. The binding energy was less than −5 kcal/mol, which indicates good binding capacity, and when the binding energy was less than −7 kcal/mol, strong binding capacity was achieved. For the molecular docking between *Caulis sinomenii* and immune‐related muscle injury, the minimum binding capacity was −9.6 kcal/mol, and the maximum binding capacity was −5.7 kcal/mol, indicating that the binding activity of the two was good, and 6 pairs with TLR4 binding ability were selected for visual docking (Figure 6B,C).

## 4. Discussion

The development of ICIs has provided a novel and revolutionary treatment option for previously incurable cancers [38]. ICIs were originally approved by the FDA for the treatment of metastatic malignant melanoma, and today, the indications for ICIs treatment have expanded to include more than 85 malignancies [39] and continue to expand. Compared with traditional chemotherapy, ICIs greatly improve the survival rate of patients [40, 41]. Moreover, ICIs are now being used early in the disease course as first‐ or second‐line treatments, either alone or in combination with chemotherapy [42]. However, this significant improvement increases systemic inflammatory responses and immune‐related effects affecting different systems, including the cardiovascular system. A 2022 meta‐analysis by Liu et al. [43] reported that CTLA‐4 inhibitor monotherapy may be associated with a higher incidence of cardiotoxicity compared with PD‐1 or PD‐L1 monotherapy. Importantly, these observations were derived from indirect comparisons across different immunotherapy regimens. In contrast, our experimental data demonstrate that combined PD‐1 and CTLA‐4 blockade was associated with greater cardiomyocyte injury than PD‐1 monotherapy, suggesting that dual ICI may further increase cardiotoxic effects compared with single‐agent PD‐1 blockade under the present experimental conditions. A systematic review and network meta‐analysis of randomized controlled trials investigating the cardiotoxicity of lung cancer–related immunotherapy versus chemotherapy confirmed that dual immunotargeted inhibitors (PD − 1 inhibitors + CTLA − 4 inhibitors) are more likely to cause adverse cardiac reactions [44]. In this work, the effects of combined PD‐1 and CTLA‐4 blockade on cardiomyocytes were studied at the cellular and animal levels. In our study, ICIs were associated with increased cardiomyocyte injury, and the combination of a PD‐1 inhibitor with a CTLA‐4 inhibitor produced greater effects than PD‐1 inhibitor monotherapy under the tested conditions. However, because the combination group in the animal experiments received a higher total antibody dose, these findings should not be interpreted as establishing a dose‐dependent relationship or a specific synergistic interaction.

At present, the mechanism of immune‐related cardiotoxicity is unclear. According to the literature, this may be related to the same or homologous antigens that cause cross‐reactivity in tumor cells and cardiomyocytes. Alternatively, increased ICIs concentrations promote the formation of autoantibodies, myosin drives cell‐mediated cytotoxicity, and high levels of cytokines recruit immune cells into the tumor microenvironment; immune tolerance, atherosclerosis, and ICIs interactions with proteins expressed in heart tissue led to complement‐mediated tissue damage. The cardioprotective effect of the PD‐1/PD‐L1 interaction is regulated by the inhibition of the production of inflammatory factors, and interfering with this interaction counteracts this protective effect, resulting in cardiotoxicity. In animal models, the inhibition of the PD‐1/PD‐L1 interaction induces the expression of multiple proinflammatory factors, including TNF‐*α* and IL‐1*β*, leading to the apoptosis of cardiomyocytes [45]. Similar conclusions were also obtained in this study, which verified that ICIs can promote the apoptosis of mouse cardiomyocytes through different detection methods at the animal level.

According to previous studies, the mechanisms of tumor‐related heart injury include the activation of the interaction between the NLRP3 inflammasome and PD‐1/PD‐L1, which is also related to inflammatory factors, and the clinical manifestations of cardiac toxicity in patients with tumors after immunotherapy are mostly explosive inflammatory responses and even inflammatory factor storms. Therefore, we speculated that NLRP3 inflammasome activation is involved in the mechanism of ICI‐related cardiotoxicity. According to the experimental data in this study, ICIs increased the degree of cardiomyocyte apoptosis and NLRP3 overexpression. On the basis of the synthesis and release of NLRP3, we detected the levels of NLRP3, ASC, and caspase‐1 and obtained the same results as expected. This finding is consistent with the results of many studies in the literature showing the mechanism of cardiotoxicity related to tumor treatment. Zhang et al. [46] reported that adriamycin‐induced cardiotoxicity could be mitigated by inhibiting the activation of the NLRP3 inflammasome. The targeting of heat shock protein mitigated doxorubicin‐induced cardiotoxicity in mice by inhibiting the NLRP3 inflammasome [47]. In addition, the literature data showed that the activation of the NLRP3 inflammasome is conducive to apoptosis, which is also consistent with our findings. On the basis of the pathway associated with NLRP3 inflammasome activation, we also detected the levels of TLR4 pathway–related proteins. The results showed that ICI treatment was associated with activation of the TLR4/MyD88/NF‐*κ*B signaling pathway and subsequently affected the expression of NLRP3 and the apoptosis of cardiomyocytes. Previous studies have shown that cyclophosphamide‐induced cardiac toxicity in mice can be improved by the TLR4/NF‐*κ*B/NLRP3 signaling pathway [48]. Adriamycin‐induced cardiotoxicity is mitigated by inhibiting the activation of the NLRP3 inflammasome [49, 50]. It can also alleviate DOX‐induced heart failure by blocking the TLR4/NF‐*κ*B pathway [51]. Studies have shown that the TLR4/NLRP3 signaling pathway plays an important role in doxorubicin‐induced cardiotoxicity in mice [52, 53]. Importantly, we selected only one cell line at this time, and it is uncertain whether ICI drugs have the same effects when applied to other cardiac muscle cell lines. In summary, we determined the increased expression of NLRP3 in AC16 cardiomyocytes and mouse cardiomyocytes by using different detection methods at different levels in vitro and in vivo. In addition, NLRP3 activation was associated with increased expression of apoptosis‐related markers in this study; however, the specific mode of cell death (apoptosis vs. pyroptosis) cannot be definitively determined based on the current data. On the basis of the findings of this study, we speculate that the TLR4/NF‐*κ*B/NLRP3 signaling pathway may be a promising therapeutic target for treating immunotherapeutically toxic hearts. Owing to the recent development of TCM, some studies have shown that TCM treatment is highly important for treating myocardial injury related to tumors.

In recent years, increasing attention has been given to the role of TCM in mitigating cardiovascular toxicity associated with anticancer therapies. *Caulis sinomenii*, a classical TCM herb, is widely recognized for its anti‐inflammatory and immunomodulatory properties and has been used in the treatment of various inflammatory and autoimmune diseases. Pharmacological studies have demonstrated that its major active component, sinomenine, can suppress inflammatory responses by inhibiting TLR4 signaling and downstream NF‐*κ*B activation, thereby reducing the production of proinflammatory cytokines. Given that ICI‐induced cardiotoxicity is characterized by excessive immune activation, inflammatory cytokine release, and cardiomyocyte injury, these properties suggest that *Caulis sinomenii* may have potential relevance in this context. Notably, in our study, activation of the TLR4–MyD88–NF‐*κ*B signaling pathway was identified as a key mechanism underlying cardiomyocyte apoptosis and inflammation induced by nivolumab combined with ipilimumab. Consistent with this mechanism, our network pharmacology and molecular docking analyses identified TLR4 as a core target and demonstrated that sinomenine exhibits strong predicted binding affinity to TLR4. These findings suggest that *Caulis sinomenii* may exert potential cardioprotective effects by modulating the TLR4/NF‐*κ*B/NLRP3 signaling axis. However, these results are based on computational predictions, and further experimental validation is required to confirm its therapeutic effects in ICI‐induced cardiotoxicity.

Next, we used network pharmacology to explore the active ingredients, targets, related signaling pathways and mechanisms of action in the treatment of immune‐related myocardial injury and molecular docking between the active ingredients and related core targets and screened a total of 6 active compounds and 33 potential disease targets for the treatment of immune‐related myocardial injury according to an OB greater than or equal to 30% and a DL greater than or equal to 0.1 and the five principles of DL. According to the analysis results, the main compounds sinomenine and *β*‐sitosterol may play key roles in the treatment of immune‐related myocardial injury. Studies have shown that *Caulis sinomenii* is mainly composed of alkaloids, triterpenoids, and volatile oils. Among them, sinomenine is the main active ingredient. Studies have shown that sinomenine can effectively inhibit the vicious cycle of inflammatory cell factors[54] and may play a therapeutic role in immune‐related myocardial injury. In this study, multiple target genes closely associated with immune‐related myocardial injury, including PTGS2, TLR4, NOS2, and others, were identified through core PPI maps. PTGS2 is involved in the synthesis of prostaglandins and promotes the inflammatory response [55]. NOS2 can further exacerbate the inflammatory response by inducing inflammatory cells to produce other proinflammatory factors, such as IL‐1*β* and TNF [56]. TLR4 also plays an important role in immune‐related myocardial injury and can activate the NF‐*κ*B pathway, a major inflammatory pathway, which in turn promotes the production of inflammatory cytokines. This molecule may be an effective target in the treatment of immune‐related myocardial injury. Through the construction of the “*Caulis sinomenii*–active ingredient–pathway–target” network and molecular docking analysis, TLR4 may be a potential target for the treatment of immune‐related myocardial injury diseases, and molecular docking revealed that the binding ability of TLR4 to the active ingredients could be as low as −7.3 kcal/mol, indicating that these active ingredients strongly interact with target targets and can form stable and effective complexes. This binding ability not only reflects the affinity between the active ingredients of *Caulis sinomenii* and the target but also predicts the possible efficacy of the drug in vivo. Through GO and KEGG pathway analyses, we found that the Th17 and NF‐*κ*B signaling pathways were the most significantly enriched, and these pathways play key roles in the pathogenesis and development of immune‐related myocardial injury. This finding is similar to the results of our previous cell and animal experiments, but we have not conducted further experimental verification of the mechanism of action of *Caulis sinomenii* in the treatment of immune‐related myocardial injury. However, our findings suggest that TCM may be an effective treatment for immune‐related myocardial injury.

Although network pharmacology and molecular docking analyses identified sinomenine as a potential TLR4‐targeting compound with favorable binding affinity, the present study did not include functional validation experiments to assess its cardioprotective effects in ICI‐induced myocardial injury models. Therefore, the therapeutic relevance of sinomenine remains exploratory. Future studies incorporating in vitro and in vivo functional assays, such as sinomenine treatment in ICI‐exposed cardiomyocytes or animal models, will be necessary to determine whether targeting TLR4 with sinomenine can effectively reverse or mitigate immunotherapy‐related cardiotoxicity. Notably, the network pharmacology and molecular docking analyses are mechanistically consistent with our experimental findings. In both in vitro and in vivo models, we identified TLR4 activation as a key upstream driver of NF‐*κ*B signaling, NLRP3 inflammasome activation, and subsequent cardiomyocyte apoptosis following ICI treatment. Consistent with this mechanism, sinomenine—one of the principal active ingredients of Caulis *sinomenii*—exhibited strong binding affinity to TLR4 in molecular docking analyses. These findings suggest that sinomenine may exert cardioprotective effects by modulating the TLR4/NF‐*κ*B/NLRP3 signaling axis, thereby potentially attenuating immunotherapy‐related myocardial injury. Although functional validation was not performed in the present study, this mechanistic overlap provides a coherent rationale linking our experimental observations with the computational prediction.

This study has a few limitations. First, the experimental design does not allow a clear distinction between the specific effects of CTLA‐4 blockade and increased total antibody exposure, as the combination group received a higher cumulative dose than the monotherapy group; therefore, it cannot be determined whether the observed differences reflect dose effects, simple additivity, or true synergistic interactions. Second, the study lacks certain essential controls, including isotype or non‐specific IgG controls and an immune‐related positive control, which may affect the specificity of the observed effects. Third, myocardial injury was primarily evaluated using histological and molecular markers without functional cardiac assessments such as echocardiography or ECG, limiting the ability to confirm functional impairment. Fourth, mechanistic interpretation is constrained by incomplete characterization of cell death pathways, as markers of pyroptosis (e.g., caspase‐1 and GSDMD) were not comprehensively assessed, making it difficult to distinguish apoptosis from inflammasome‐mediated pyroptosis. Fifth, the in vitro experiments were conducted in a single cardiomyocyte cell line (AC16), which may limit generalizability to other cardiac cell types or in vivo conditions. Finally, although network pharmacology and molecular docking analyses suggested that *Caulis sinomenii* and its active component sinomenine may target the TLR4/NF‐*κ*B/NLRP3 pathway, these findings are based on computational predictions and were not validated through functional experiments. Future studies incorporating dose‐matched designs, additional controls, multi‐model validation, and functional assays are needed to further clarify the mechanisms and therapeutic implications of ICI‐related cardiotoxicity.

## 5. Conclusion

In recent years, advances in tumor diagnosis and treatment, particularly genomics‐guided precision therapy, have significantly prolonged patient survival, transforming cancer into a chronic disease in many cases [57]. With the widespread application of ICIs, patient prognosis has improved; however, immunotherapy‐related cardiotoxicity has also become an important clinical concern. In this study, nivolumab, particularly when combined with ipilimumab, was associated with increased cardiomyocyte injury and activation of the TLR4–MyD88–NF‐*κ*B–NLRP3 signaling pathway under the present experimental conditions. Knockdown of TLR4 or NLRP3 attenuated these changes, supporting the involvement of this signaling axis in ICI‐related myocardial injury. Network pharmacology and molecular docking analyses further identified TLR4 as a potential target and suggested that active components of *Caulis sinomenii*, particularly sinomenine, may modulate this pathway. However, because the combination group received a higher total antibody dose than the monotherapy group, the present study does not establish a dose‐dependent relationship or distinguish between increased total drug exposure and the specific contribution of CTLA‐4 blockade. Overall, these findings provide mechanistic insight into ICI‐related cardiotoxicity and support further investigation of the TLR4–MyD88–NF‐*κ*B–NLRP3 axis as a potential therapeutic target.

**Nomenclature**
PTGS2: prostaglandin‐endoperoxide Synthase 2
TLR4: toll‐like Receptor 4
STAT3: signal transducer and activator of transcription 3
IDO1: indoleamine 2,3‐dioxygenase 1
NOS2: nitric oxide synthase 2
IL‐5: Interleukin 5
mTOR: mammalian target of rapamycin
LCK: lymphocyte‐specific protein tyrosine kinase
BTK: Bruton tyrosine kinase
FYN: FYN proto‐oncogene, Src family tyrosine kinase

## Author Contributions

Mengjiao Gao: writing—designed the research plan and specific content, conducted the research, curated the data, and wrote the papers. Xinya Liu: software, designed the research, and analysis. Li Wu: participated in the research and supervised the thesis. Yun Jiang: supervision and investigation. Jian Yu: methodology and formal analysis. Yuanming Zhang: writing—review and editing, supervised the experimental scheme, and final version

## Funding

This study was supported by the Graduate Student Innovation Project Fund of Autonomous Region (No.XJ2023G181) awarded to Mengjiao Gao. Key Laboratory of High Incidence Disease Research in Xingjiang Ministry of Education (Open Project–Young Researcher Program), (No.2023C03) awarded to Mengjiao Gao. This study was supported by the Affiliated Cancer Hospital of Xinjiang Medical University, Xinjiang Key Laboratory of Oncology (No.XJKLO‐2025S005) awarded to Mengjiao Gao and Tianshan Talents–Leading Technological Innovation Leaders (No. 2022TSYCLJ0068) awarded to Yuanming Zhang.

## Ethics Statement

All experimental protocols involving animal models were reviewed and approved by the Animal Testing Ethics Committee of Xinjiang Medical University Affiliated Tumor Hospital (The Third Clinical Medical College), Xinjiang, Urumqi City, China (Ethics Approval Number: MDL2023‐10‐20‐03). All animal procedures were performed in compliance with the Guide for the Care and Use of Laboratory Animals (National Institutes of Health, United States), and efforts were made to minimize animal suffering. Male BALB/c mice were maintained under pathogen‐free conditions with controlled temperature, humidity, and a 12‐hour light–dark cycle, with ad libitum access to food and water.

## Consent

The authors have nothing to report.

## Conflicts of Interest

The authors declare no conflicts of interest.

## Data Availability

The data that support the findings of this study are available from the corresponding author upon reasonable request.
